# Evaluation of Lumbar Sonography as a Learning Aid for Performing Subarachnoid Block Using the Paramedian Approach by Medical Junior Resident Anaesthesiologists: A Randomized Controlled Trial

**DOI:** 10.7759/cureus.38871

**Published:** 2023-05-11

**Authors:** Yashaswini Gorle, Sujatha Munireddy Papireddy, Sumanth Tarigonda

**Affiliations:** 1 Anaesthesia, Sri Devaraj Urs Medical College, Kolar, IND

**Keywords:** ultrasound-guided, difficult spinal, spinal anaestheia, paramedian, pre-procedural ultrasound

## Abstract

Background: Pre-procedural ultrasound can be used to identify the subarachnoid space in difficult spinal procedures. However, multiple punctures can result in numerous complications, including post-dural puncture headache, neural trauma, and spinal and epidural haematoma. Thus, the following hypothesis was proposed: in contrast to the conventional blind paramedian dural puncture, pre-procedural ultrasound results in a successful dural puncture on the first attempt.

Methods: In this prospective, randomised controlled study, 150 consenting patients were randomly assigned to one of the two groups: ultrasound-guided paramedian (UG) and conventional blind paramedian (PG). In the UG paramedian group, pre-procedural ultrasound was performed to mark the insertion site, whereas, in the PG group, the landmark technique was used. A total of 22 different anaesthesiology residents performed all subarachnoid blocks.

Results: The time taken to perform spinal anaesthesia in the UG group was 38-49.5 s, which is shorter than the time taken in the PG group, which was 38-55 s, with a p-value < 0.046, which is statistically significant. The primary outcome of a successful dural puncture on the first attempt was not significantly higher in the UG group (49.33%) than in the PG group (34.67%), with a p-value < 0.068. The number of attempts taken for a successful spinal tap in the UG group was a median of 2.0 (1 to 2), and the PG group had a median of 2 (1 to 2.5), with a p-value < 0.096, which is statistically non-significant.

Conclusion: Ultrasound guidance showed improvement in the success rate of paramedian anaesthesia. In addition, it improves the success rate of dural puncture and the rate of puncture on the first attempt. It also shortens the time required for a dural puncture. In the general population, the pre-procedural UG paramedian group did not outperform the PG paramedian group.

## Introduction

The subarachnoid block is appropriate for many surgical procedures, such as abdominal gynaecological procedures, obstetric anaesthesia, caesarean section, hip and knee joint replacements, hernia repairs, perineal surgery, and prostatectomy. In individuals with obesity, prior spinal surgery, spinal deformity, and degenerative changes brought on by ageing, the technical difficulty of neuraxial blockade corresponds with the quality of perceptible surface landmarks [1,2].

The paramedian approach is an equally good alternative, as it is less inclined to be affected by the osteoarthritic changes in the spine [3-5]. In the paramedian approach, less resistance can be encountered, as supraspinous and interspinous ligaments are not commonly located.

Ultrasound can be utilized to improvise the success rate of dural puncture and has been reported for a long time, and more frequently in recent studies, especially in the subset of patients where the difficult dural puncture is predicted, including in obese as well as elderly patients [6]. Both pre-procedural ultrasound imaging and real-time ultrasound-guided spinal needle insertion by trained anesthesiologists have enhanced spinal-anaesthesia success rates. In the ultrasound, the bone casts a dense, acoustic shadow as the ultrasound does not penetrate the bone. The interpretation of lumbar spine anatomy in ultrasound is based on the typical acoustic shadows due to the contours created by the bony surfaces on the lumbar vertebra, especially the posterior surface. Ultrasound can be helpful in the facilitation of neuraxial block in subjects with difficult palpatory landmarks, such as obese patients, elderly patients, and those who have anatomical abnormalities in the vertebrae. The technique used to administer spinal anaesthesia, the needle size, and the design of the needle all influence how rapidly post-dural puncture headache arises [7].

The primary objectives of this study were to determine whether a pre-procedural lumbar ultrasound scan improves the rate of first needle insertion attempt success while performing subarachnoid block by resident anesthesiologists when compared to a landmark-guided approach. The secondary objectives of this study were the number of attempts and needle redirections to perform subarachnoid block and the total amount of time taken in seconds or minutes to perform the subarachnoid block and complications such as bloody tap, paraesthesias, failed or inadequate block.

We conducted this prospective study to identify the efficacy of pre-procedural ultrasound-guided paramedian in comparison to conventional blind paramedian subarachnoid block (SAB). This article was presented as a paper at the Indian Society of Anaesthesiologists Conference (ISACON), Shillong on November 26, 2022.

## Materials and methods

This study was approved by the Institutional Ethics Committee NO. SDUMC /KLR/IEC/615/2020-21. This study consisted of patients admitted for surgical procedures under spinal anaesthesia. Informed consent was obtained from the patients and residents participating in the randomized controlled trial.

Inclusion criteria

Patients among the age group 18 to 70 years old with an ASA (American Society of Anesthesiologists) physical status of 1 or 2 undergoing abdominal surgeries and lower limb procedures under spinal anaesthesia were included in the study. Anaesthesiology residents who have almost completed one year of the residency program participated in this study. This was conducted in RL Jalappa Hospital, Tamaka, in the Indian state of Karnataka.

Exclusion criteria

Patients who refused to be included in the following study were excluded. The following exclusion criteria were also applied: patients contraindicated for spinal anaesthesia due to an infection at the site of injection, hypovolemia, allergy to local anaesthetics, raised intracranial pressure, coagulopathies, and fixed cardiac output conditions. Moreover, the anaesthesiology residents excluded from the study were those who were already proficient with the paramedian approach to subarachnoid block and those who have already performed the procedure more than 10 times. 

Study interventions 

Group Ultrasound Guidance (UG)

**Figure 1:**
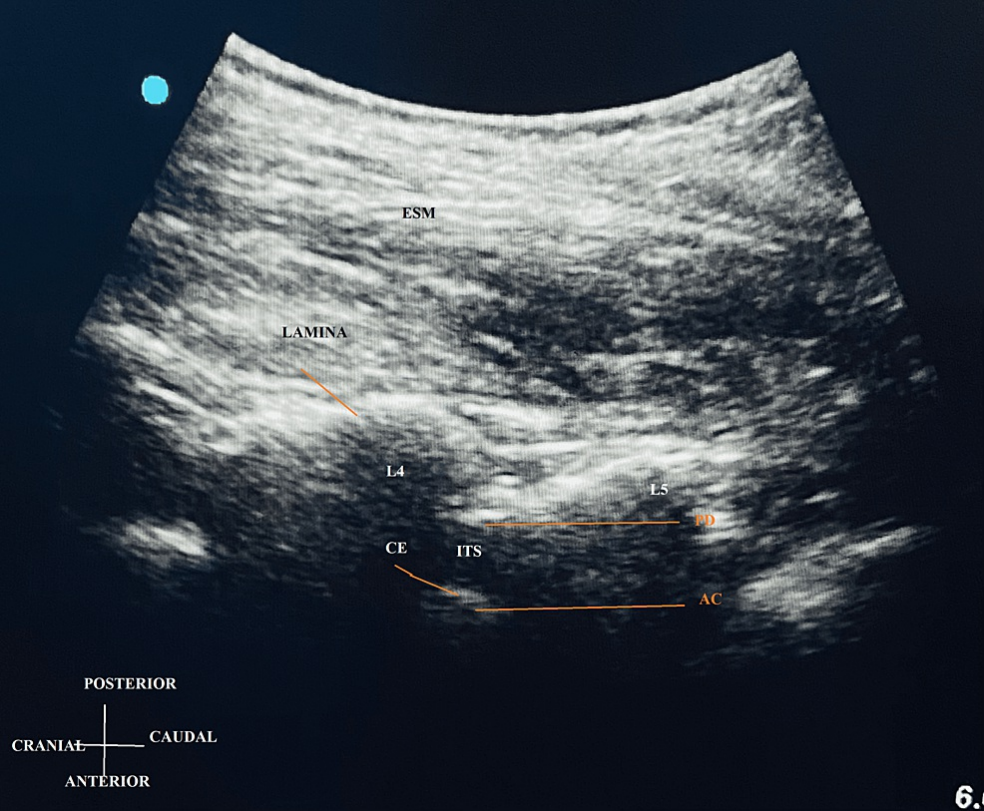
Paramedian Sagittal Oblique View AC: anterior complex; CE: cauda equina; ESM: erector spinae muscle; ITS: intrathecal space; PD: posterior dura

Residents in this group were taught how to use a paramedian sagittal oblique plane to locate the L3-L4 intervertebral space and visualize the intrathecal space between the ligamentum flavum and the dura mater complex and the posterior longitudinal ligament (Figure 1). The sacrum was identified, and the interlaminar space between L5 and S1 was found after getting a paramedian sagittal oblique image of the neuraxis. Furthermore, interlaminar spaces were counted from below upward as each interlaminar space corresponds to the following laminar spaces. The point of needle insertion was indicated on the surface, and the needle’s direction was recorded. The interspinous area, which clearly shows the anterior (ligamentum flavum dura complex) and posterior (posterior longitudinal complex), was noted.

Group Palpation Guidance (PG)

**Figure 2:**
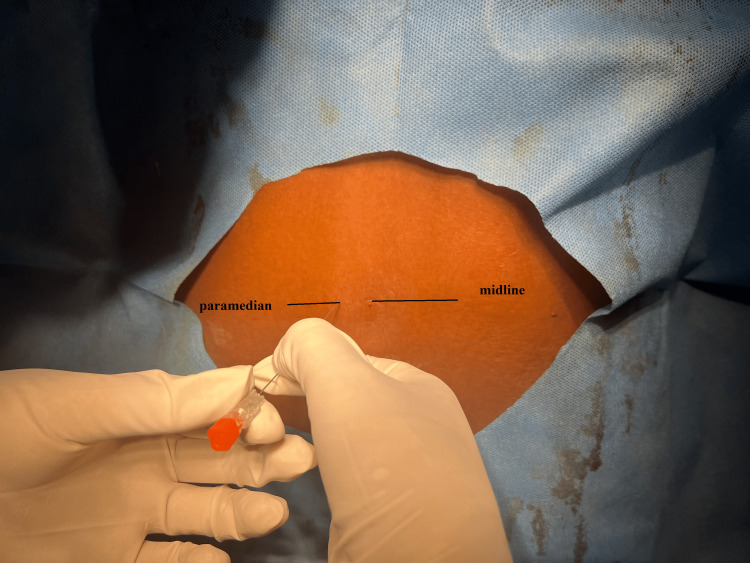
Paramedian Blind Spinal

In this group, using didactic lectures, the residents were taught about the performance of paramedian subarachnoid block using palpation of landmarks. After confirming the correct interspinous space, the needle was then inserted 1 cm laterally and inferior to the corresponding interspace, which goes cephalic and medially angled (Figure 2). In order to extract cerebrospinal fluid, the needle was moved toward the cephalic direction after making contact with the lamina and then progressively advanced into the subarachnoid area; we adapted this procedure from the methodology set out by Srinivasan et al. [4].

After performing immediate pre-anaesthetic evaluation and shifting the patient onto the operation table, standard monitoring of non-invasive blood pressure, five-electrode electrocardiogram, and oxygen saturation were documented and intravenous access was secured. Patients were made to sit in a comfortable position in preparation for the preprocedural ultrasound prior to doing markings for the paramedian; surface marking was performed under the direct supervision of a consultant anaesthesiologist. Strict asepsis was maintained while performing the subarachnoid block. Skin infiltration with 2% lignocaine was done at the point of needle insertion. A 25 G Quincke-Babcock spinal needle was used to perform the block using any of the techniques mentioned above based on group allocation. After confirming the correct position of the needle by aspirating clear cerebrospinal fluid, a pre-determined dose of 0.5% Bupivacaine Heavy was injected, the patient was positioned supine, and the level of block achieved was noted.

Outcome 

The primary outcome variable was a successful dural puncture on the first attempt. The secondary outcome variable consists of the number of needle attempts required for a successful dural puncture (a subsequent needle attempt is described as the complete withdrawal of the spinal needle from the following structures followed by reinsertion), the number of needle redirections (a needle redirection is described as a partial withdrawal of the needle from the patient's skin as well as a shift in the needle's course of insertion) and the total amount of time taken to perform the spinal anaesthesia (identified as the time from the first insertion of the spinal needle until the withdrawal of the spinal needle after intrathecal injection of the anaesthetic solution).

Sampling size and sampling method

A randomised prospective single-blinded study was done on 150 patients. A total of 150 patients who were scheduled for lower abdominal and lower limb surgeries were randomly allocated into two groups ultrasound-guided paramedian group (UG) and a paramedian blind group (PG). Patients were categorized into two groups. A total of 22 residents were divided into two groups at random, with each resident performing spinal anaesthesia on five patients. The residents chosen were junior residents who completed one year of residency and were proficient in giving subarachnoid blocks through a midline approach. They were instructed about the paramedian spinal procedures using mannequins and live demonstrations for both groups.

Randomisation

Computer-generated block randomisation was done to randomize the patients into two different procedural groups. Due to the nature of the study, only patients were blinded towards the study; residents could not be blinded towards the study.

Statistics 

The major outcome variables were dural puncture, number of attempts, number of needle directions, time taken, success, and bloody tap. The study group (ultrasound group vs. palpation group) was considered the primary explanatory variable. Socio-demographic data and clinical variables formed the group of explanatory variables. The main result was a dural puncture that performed well on the first attempt.

The chi-square test was used for inferential analysis. When necessary, Fisher’s exact test was applied. For data that do not follow a normal distribution-like number of attempts, number of redirections, and pain scores-descriptive characteristics were expressed as medians with interquartile range. Inferential analysis was done with the Mann-Whitney U test. For data that are continuous, like the time needed to perform spinal anaesthesia, descriptive characteristics were expressed as mean ± standard deviation. Analysis of variance and independent sample t-tests was performed. 

## Results

In our study, 150 patients posted for surgery under spinal anaesthesia were allocated into two groups, Group UG (ultrasound paramedian) and Group PG (paramedian blind). The following are the results obtained after statistical analysis. Twenty-two residents did five procedures each in their respective allotted groups. Randomisation of 150 patients was done. There were no exclusion criteria as all the patients met the criteria, and difficulties were resolved. No patients were lost to follow-up. None had scoliosis. The study was conducted over a period of three years. The trial was stopped as an adequate sample size was achieved. The two groups had almost similar baseline demographic and types of surgery given in Table 1.

**Table 1:** Demographic and baseline characteristics

	Group PG	Group UG
Age	51.69 ± 15.23	51.69 ± 15.23
Gender		
Male	48	41
Female	27	34
Weight	84.63 ± 16.09	70 ± 11.13
Type of Surgery		
General Surgery	25	29
Gynaecology	16	18
Orthopaedics	34	28

Baseline data

**Figure 3:**
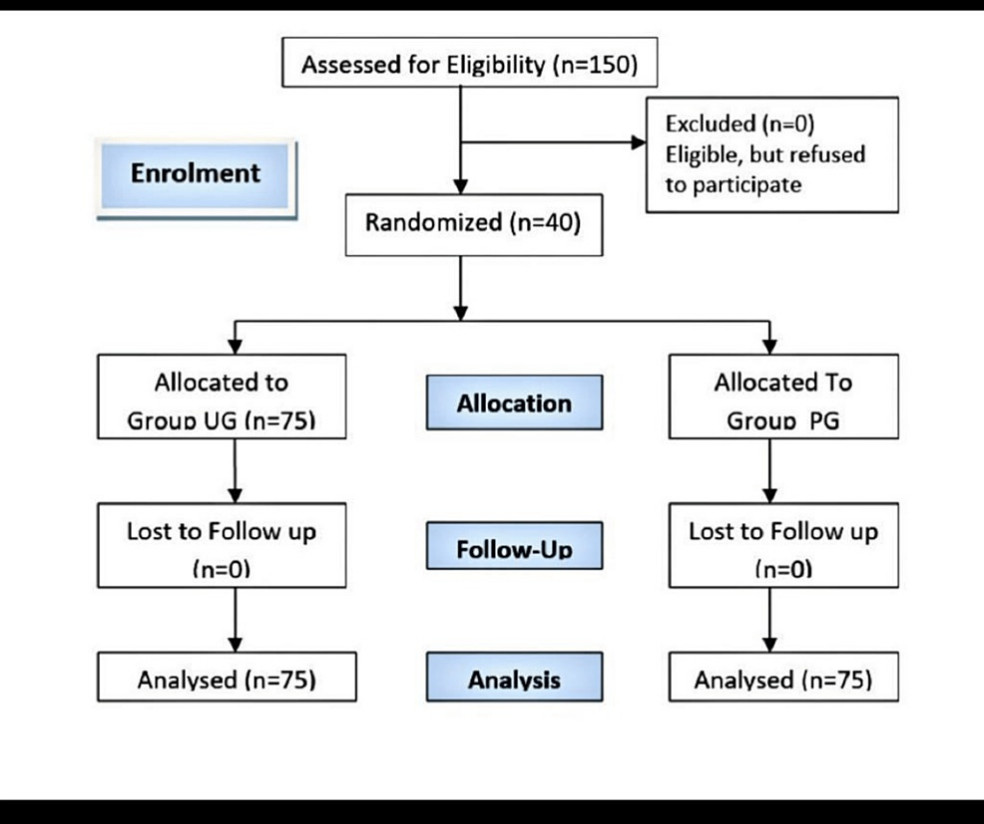
Consolidated Standards of Reporting Trials (CONSORT) flow diagram Group UG: Ultrasound-guided paramedian spinal; Group PG: Blind paramedian spinal

The data was assessed using CONSORT (Figure 3). 

Outcome and estimation

Among those that belong to Group UG, 37 (49.33%) participants were successfully administered with a dural puncture on the first attempt. On the other hand, among those in Group PG, 26 (34.67%) participants were successfully administered with a dural puncture on the first attempt (Table 2). There is no statistical difference between the two groups with respect to the successful dural puncture on the first attempt.

**Table 2:** Comparison of successful dural puncture in the first attempt Group UG: Ultrasound-guided paramedian spinal; Group PG: Blind paramedian spinal

Dural Puncture	Study Group		Chi-square value	p-value
	Group UG (N = 75)	Group PG (N = 75)		
Yes	37 (49.33%)	26 (34.67%)	3.31	0.0688
No	38 (50.67%)	49 (65.33%)		

The total number of attempts in Group UG was around the median of 2 with an interquartile range of 1.0 to 2.0. In Group PG, the median value was 2 with an interquartile range of 1.0 to 2.5. The p-value of 0.098 was obtained with respect to the total number of attempts, which is statistically not significant (Table 3). 

**Table 3:** Comparison of the total no. of attempts among the study groups (N = 150) IQR: interquartile range

Parameter	Total No. of Attempts	Mann-Whitney U test (p-value)
	Median (IQR)	
UG (N = 75)	2.00 (1.0 to 2.0)	0.0908
PG (N= 75)	2.00 (1.0 to 2.5)	

In Group UG, the total number of redirections where around the median of 2.00 with an interquartile range of 2.0 to 2.0. In Group PG, the median was 2.00 with an interquartile range of 1.0 to 2.0. A p-value of 0.0095 was obtained, which is statistically significant. Group PG has fewer redirections when compared to Group UG (Table 4).

**Table 4:** Comparison of the total no. of redirections among study groups (N = 150) IQR: interquartile range

Parameter	Total No. of Redirections	Mann-Whitney U test (p-value)
	Median (IQR)	
UG (N = 75)	2.00 (2.0 to 2.0)	0.0095
PG (N = 75)	2.00 (1.0 to 2.0)	

In Group UG, the time for spinal anaesthesia was around 45.00 secs (38.0 to 49.5), and in Group PG, the median value was 48.00 secs (38.0 to 55.0) with a p-value of 0.0464, which is statistically significant. Group UG has taken less time when spinal anaesthesia was performed on its participants (Table 5). 

**Table 5:** . Comparison of time for SA among study groups (N = 150)

Parameter	Time for SA	Mann-Whitney U Test (p-value)
	Median (IQR) (secs)	
UG (N = 75)	45.00 (38.0 to 49.5)	0.0464
PG (N = 75)	48.00 (38.0 to 55.0)	

Harms

More complications (bloody tap, paresthesia) occurred in the PG group compared to the UG group and patients were followed for 24 hours according to the hospital protocol. These complications are associated with the study.

## Discussion

Generalisability and interpretation

The present study was conducted to determine whether a preprocedural lumbar ultrasound scan can improve the success rate of the first needle insertion attempt while performing a subarachnoid block in comparison to the landmark-guided approach. The objectives of the following study were similar to that of the studies done by Chong et al. [8], Creaney et al. [9], Plewa et al. [10], Perlas et al. [11], Turkstra et al. [12], Sahin et al. [13], Lim et al. [14], Kalagara et al [15].

A total of 150 subjects who are undergoing surgeries of the lower abdomen and lower limbs under spinal anaesthesia were included in the present study. Seventy-five subjects were recruited into Group UG (USG-guided group): USG group) and 75 subjects into Group PG (palpation-guided group). The studies conducted by Lim et al. [14], Creaney et al. [9], and Sahin et al. [13] included pregnant women scheduled for caesarean section. Chong et al. [8] conducted real-time ultrasound. Kalagara et al. [15] and Chin et al. [17] studied 120 orthopaedic subjects, while Chong et al. [8] studied 60 subjects aged between 18 to 75 years with a body mass index (BMI) of ≤ 30 kg/m2. In the study by Chin et al. [17], they included subjects with a BMI greater than 35 kg/m2 and spinous processes, which were poorly palpable. Sahin et al. [13] did their study on 100 parturients by including 50 thin subjects (BMI < 30 kg/m2), and the other half comprised 50 obese subjects (BMI ≥ 30 kg/m2). Chin et al. [17], did their study in Canada, Chong et al. [8] in Malaysia, Sahin et al. [13] in Turkey, Lim et al. [14] in China, and Creaney et al. [9] in Ireland.

The gender distribution was comparable between the groups, with 54.67% males in the USG-guided group compared to 64% males in the palpation-guided group. But there was a significant difference in the mean age between the groups. The mean age in the PG group (51.69 years) was significantly higher than the mean age in the USG group (41.39 years). In the present study, in the USG-guided group, half of the subjects (37 subjects) had a successful dural puncture (49.3%) while in the control group, 34.6% (26/75 subjects) had a successful dural puncture. Multiple attempts at successful dural punctures with a single needle pass are related to an increased risk of consequences, such as post-dural puncture headache, paresthesia, and epidural hematomas. In the USG-guided group, 49.33% had a successful first needle insertion/dural puncture on the first attempt, but in the control group, only 34.67% had reported success on the first attempt. But this difference was not statistically significant: 90.67% required verbal assistance in the palpation-guided control group compared to only 82.67% in USG (ultrasound) guided group. The findings of the present study were similar to the other studies reported in the past, substantiating the use of USG-guided SAB (subarachnoid block).

In the paramedian approach, the amount of resistance encountered is small, as supraspinous ligaments and interspinous ligaments are not encountered commonly. Of the subjects, 65.33% did not have a successful dural puncture in the first attempt in the palpation-guided control group. 

The median number of attempts was 2 with an IQR (interquartile range) of 1 to 2 in the USG-guided group compared to the two attempts with an IQR of 1 to 2.5 in the control group. There was no significant difference in the median number of attempts between the groups. Successful first attempts were higher in the USG than in the control group (87% vs. 43%, P < 0.001) in the study by Chong et al. [8]. Additionally, they noticed that the USG group had a greater rate of successful single-needle passes (47% vs. 20%, P = 0.028). Identical reports were reported by Conroy et al. [16] where a successful first attempt was higher in the UG Group compared with the PG Group (87 vs. 43).

The use of USG in the study by Creaney et al. [9] decreased the number of needle passes without increasing the procedure’s overall duration. Sahin et al. [13] found in their study that the ultrasonography group had fewer attempts for puncture. According to the study by Lim et al. [14], the ultrasonography group had significantly better first-attempt success rates than the control group (87.5% vs. 52.5%, P = .001), with fewer cases requiring > 10 needle passes (1 vs. 17, P = .001), and with fewer skin punctures and needle passes (P = .001 for both) than the control group.

Srinivasan et al. [4] conducted a study with the following results: the number of needle attempts noted in the UG Group was around 8.3, and in comparison, the conventional midline group, Group C, is at 4. This difference was notably significant [4]. Three groups were compared by Rizk et al. [5]. The primary outcome showed that successful dural puncture was higher in groups LM (landmark) at 77%, followed by UM (ultrasound midline) at 73%, and UP (ultrasound paramedian) at around 42%. The p-value was statistically significant. The US technique required fewer attempts and fewer needle attempts to enter the subarachnoid space, according to Srinivasan et al. [4] when making comparisons of the paramedian vs. palpation at the midline approach for an elderly orthopaedic group. A similar study conducted by Turkstra et al. [12] concluded that there is not much additional advantage in using preprocedural ultrasound by junior residents in doing spinal anaesthesia.

In the present study, the median time taken in administering spinal anaesthesia was less in the USG-guided group (45 seconds) compared to the palpation-guided control group (48 seconds). It was statistically significant that the groups had a median difference of three seconds. In the study conducted by Chong et al. [8], the UG Group took 0.69 sec less time for dural puncture than the PG Group, which took 1.60 min; here, the p-value was statistically significant. This was consistent with the findings of Conroy et al. [16]. They observed a time of 1.2 minutes with a range of 0.2 to 15 minutes. A study conducted by Rizk et al. [5] concluded that the time taken to perform the spinal anaesthesia was not different between the LM and UM groups (87.24 ± 79.51 s and 116.32 ± 98.12 s, respectively) but shorter than in group UP (154.58 ± 91.51 s; P < 0.001).

Patient satisfaction 

The level of satisfaction of the patients was rated as good by 48% in USG-guided (UG) group compared to only one subject (1.33%) rating as good in the palpation-guided (PG) control group in the present study. The difficulty in the administration of spinal anaesthesia has a correlation with the palpability of the surface landmarks. These landmarks may get distorted in subjects who are obese, subjects with a previous history of spine surgeries, subjects with spine deformities, and subjects having degenerative changes, which are associated with ageing, Sprung et al. [1], de Filho et al. [2]. Lim et al. [14], in their study, observed that the satisfaction scores of the subjects increased significantly in the USG group (p-value of 0.001). There was no statistical difference in the study conducted by Turkstra et al. [12], in patient satisfaction scores.

Periprocedural pain 

It was analysed by VAS (visual analogue scale score) in the present study. There was a significant difference in median peri-procedural pain between the groups with a higher VAS score of 4 in the USG group compared to only 3 in the PG Group.

Srinivasan et al. [4] conducted a study on landmark- and ultrasound-guided paramedian in which VAS pain scores were similar in two groups and were statistically insignificant. However, there was no statistical difference in the study conducted by Turkstra et al. [12]. With regard to pre-procedural VAS scores, the pre-procedural pain score was higher in UP than in the other two groups as the needle has to pierce the erector spinal muscle, according to Rizk et al. [5].

Complications

The frequency of successful needle manipulations is one of the key metrics used to quantify the technical challenges associated with the administration of spinal anaesthesia. Complications such as post-dural puncture headache, paresthesia, and epidural hematoma have been linked to an increase in the number of attempts [2-3]. The optimal method for administering spinal anaesthesia is a successful dural puncture with a single needle pass because additional attempts raise the risk of complications. Kalagara et al. [15] in their review article observed that based on recent evidence, the use of neuraxial USG before the procedure can reduce the rate of occurrence of complications like headache, puncture of the vessels, and back pain.

Bloody tap

In Group UG, complications involved bloody tap 13 (17.3%), and in Group PG, the score given for bloody tap was 14 (18.67%), which is statistically not significant. There was no statistical difference in the study conducted by Turkstra et al. [12] in bloody tap. An increase in the number of attempts in the UP Group had more incidence of bloody tap, according to the study conducted by Rizk et al. [5].

Paresthesia

No paresthesia was noted in the UG Group, whereas in the PG Group, 2.65% of patients had paresthesia. Paresthesia elicitation is a substantial risk factor for permanent neurologic impairment following spinal anaesthesia [3].

Limitations

The lack of blinding of residents and observers collecting the data caused bias. Operator bias and patient bias were not completely eliminated. The USG-guided technique also has its limitations. In the populations intended for USG-guided dural puncture, there can be restrictions in imaging the vertebral canal on USG. Structures such as the ligamentum flavum, dura mater, and the posterior portion of the vertebral body may be difficult to see. Because of the attenuation that occurs when ultrasound travels a greater distance in soft tissue, identifying structures in obese patients can be challenging. Because of the varying speeds in adipose layers which are irregularly shaped, a phase aberration effect can also occur. Clinical-demographic conditions of the patient, thoraco-lumbar spine surfaces, and anatomy-related data were not collected. Positioning difficulty and marking difficulty data are unavailable.

## Conclusions

Ultrasound guidance showed improvement in the success rate of paramedian anaesthesia. In addition, it improves the success rate of dural puncture and the rate of puncture on the first attempt. It also shortens the time required for a dural puncture. In the general population, pre-procedural UG paramedian group did not outperform PG paramedian group.

Spinal anaesthesia is chiefly considered a blind procedure. Ultrasound can be used to identify the better point of needle insertion and better angulation. The advantages of pre-puncture ultrasonic scanning outweigh the disadvantages of its lengthier total duration. It has proven beneficial in elderly patients with hip fractures and spine deformities like scoliosis.

## References

[1] Sprung J, Bourke DL, Grass J, Hammel J, Mascha E, Thomas P, Tubin I (1999). Predicting the difficult neuraxial block: a prospective study. Anesth Analg.

[2] de Filho GR, Gomes HP, da Fonseca MH, Hoffman JC, Pederneiras SG, Garcia JH (2002). Predictors of successful neuraxial block: a prospective study. Eur J Anaesthesiol.

[3] Wulf H (1996). Epidural anaesthesia and spinal haematoma. Can J Anaesth.

[4] Kallidaikurichi Srinivasan K, Iohom G, Loughnane F, Lee PJ (2015). Conventional landmark-guided midline versus preprocedure ultrasound-guided paramedian techniques in spinal anesthesia. Anesth Analg.

[5] Rizk MS, Zeeni CA, Bouez JN, Bteich NJ, Sayyid SK, Alfahel WS, Siddik-Sayyid SM (2019). Preprocedural ultrasound versus landmark techniques for spinal anesthesia performed by novice residents in elderly: a randomized controlled trial. BMC Anesthesiol.

[6] Grau T, Leipold RW, Horter J, Conradi R, Martin E, Motsch J (2001). The lumbar epidural space in pregnancy: visualization by ultrasonography. Br J Anaesth.

[7] Kwak KH (2017). Postdural puncture headache. Korean J Anesthesiol.

[8] Chong SE, Mohd Nikman A, Saedah A, Wan Mohd Nazaruddin WH, Kueh YC, Lim JA, Shamsul Kamalrujan H (2017). Real-time ultrasound-guided paramedian spinal anaesthesia: evaluation of the efficacy and the success rate of single needle pass. Br J Anaesth.

[9] Creaney M, Mullane D, Casby C, Tan T (2016). Ultrasound to identify the lumbar space in women with impalpable bony landmarks presenting for elective caesarean delivery under spinal anaesthesia: a randomised trial. Int J Obstet Anesth.

[10] Plewa MC, McAllister RK (2022). Postdural puncture headache. StatPearls.

[11] Perlas A, Chaparro LE, Chin KJ (2016). Lumbar neuraxial ultrasound for spinal and epidural anesthesia: a systematic review and meta-analysis. Reg Anesth Pain Med.

[12] Turkstra TP, Marmai KL, Armstrong KP, Kumar K, Singh SI (2017). Preprocedural ultrasound assessment does not improve trainee performance of spinal anesthesia for obstetrical patients: a randomized controlled trial. J Clin Anesth.

[13] Sahin T, Balaban O, Sahin L, Solak M, Toker K (2014). A randomized controlled trial of preinsertion ultrasound guidance for spinal anaesthesia in pregnancy: outcomes among obese and lean parturients: ultrasound for spinal anesthesia in pregnancy. J Anesth.

[14] Lim YC, Choo CY, Tan KT (2014). A randomised controlled trial of ultrasound-assisted spinal anaesthesia. Anaesth Intensive Care.

[15] Kalagara H, Nair H, Kolli S (2021). Ultrasound imaging of the spine for central neuraxial blockade: a technical description and evidence update. Curr Anesthesiol Rep.

[16] Conroy PH, Luyet C, McCartney CJ, McHardy PG (2013). Real-time ultrasound-guided spinal anaesthesia: a prospective observational study of a new approach. Anesthesiol Res Pract.

[17] Chin KJ, Perlas A, Chan V, Brown-Shreves D, Koshkin A, Vaishnav V (2011). Ultrasound imaging facilitates spinal anesthesia in adults with difficult surface anatomic landmarks. Anesthesiology.

